# Oriented and Ordered Biomimetic Remineralization of the Surface of Demineralized Dental Enamel Using HAP@ACP Nanoparticles Guided by Glycine

**DOI:** 10.1038/srep40701

**Published:** 2017-01-12

**Authors:** Haorong Wang, Zuohui Xiao, Jie Yang, Danyang Lu, Anil Kishen, Yanqiu Li, Zhen Chen, Kehua Que, Qian Zhang, Xuliang Deng, Xiaoping Yang, Qing Cai, Ning Chen, Changhong Cong, Binbin Guan, Ting Li, Xu Zhang

**Affiliations:** 1 School of Stomatology, Hospital of Stomatology, Tianjin Medical University, 12 Observatory Road, Tianjin 300070, P.R. China; 2 Discipline of Endodontics, Faculty of Dentistry, University of Toronto, 27 King’s College Cir, Toronto ON M5S, Canada; 3 Department of Geriatric Dentistry, Peking University School and Hospital of Stomatology, 22 Zhongguancun South Street, Beijing 100081, P.R. China; 4 The Key Laboratory of Beijing City on Preparation and Processing of Novel Polymer, Beijing University of Chemical Technology, 15 North Three-ring East Road, Beijing 100029, P.R. China; 5 Academic Committee of Bybo dental group, 4 Qinian Main Street, Beijing 100010, P.R. China

## Abstract

Achieving oriented and ordered remineralization on the surface of demineralized dental enamel, thereby restoring the satisfactory mechanical properties approaching those of sound enamel, is still a challenge for dentists. To mimic the natural biomineralization approach for enamel remineralization, the biological process of enamel development proteins, such as amelogenin, was simulated in this study. In this work, carboxymethyl chitosan (CMC) conjugated with alendronate (ALN) was applied to stabilize amorphous calcium phosphate (ACP) to form CMC/ACP nanoparticles. Sodium hypochlorite (NaClO) functioned as the protease which decompose amelogenin *in vivo* to degrade the CMC-ALN matrix and generate HAP@ACP core-shell nanoparticles. Finally, when guided by 10 mM glycine (Gly), HAP@ACP nanoparticles can arrange orderly and subsequently transform from an amorphous phase to well-ordered rod-like apatite crystals to achieve oriented and ordered biomimetic remineralization on acid-etched enamel surfaces. This biomimetic remineralization process is achieved through the oriented attachment (OA) of nanoparticles based on non-classical crystallization theory. These results indicate that finding and developing analogues of natural proteins such as amelogenin involved in the biomineralization by natural macromolecular polymers and imitating the process of biomineralization would be an effective strategy for enamel remineralization. Furthermore, this method represents a promising method for the management of early caries in minimal invasive dentistry (MID).

Dental caries begin at the outermost layer of teeth (enamel) with the damage of dental hard tissues. This process is called demineralization, and it is attributed to the loss of mineral ions from the hydroxyapatite (HAP) lattice by organic acids produced by bacteria on the surface of enamel. It has been established that dental caries are a dynamic disease process caused by the imbalance between the demineralization and remineralization processes^1^,^2^. The latter process in which mineral ions are returned to the lattice of minerals or in which new crystals form *in situ* in the lesions^3^. Thus, if this balance is not restored by early intervention measures, including remineralization treatments and dental plaque control, caries are likely to gradually develop from enamel to dentin, leading to tooth cavities and eventually the loss of teeth. Therefore, the process has considerable clinical significance in the prevention and treatment of early stages of dental caries disease and is thus regarded as one of the important treatment technologies in minimally invasive dentistry (MID)^4^.

According to the principle of MID, the non-invasive remineralizing treatment is more acceptable than the common filling treatment during the early stages of dental caries disease^5^. To date, it has been well documented that fluoride treatment remains the best remineralizing method for early enamel caries^6^,^7^,^8^. The remineralization of teeth with fluoride is achieved through the formation of fluorapatite based on the epitaxial growth of the residual crystals^9^,^10^,^11^. However, due to the lack of ability to guide the formation of mineral crystals, it is difficult for fluoride to result in oriented and ordered mineral crystals on the surface of enamel under physiological conditions^12^. The mineral crystals in mature enamel are highly elongated and oriented prisms of carbonated hydroxyapatite, which is essential for the mechanical properties of enamel^12^,^13^. Thus, an ideal mineralizing material should achieve the organization and micro-architecture of mineral crystals that mimic natural ones to the greatest extent possible.

Hopefully, with the progress in the understanding of the biomineralization of dental hard tissues^14^,^15^,^16^, it will become possible to develop a biomimetic remineralization strategy by simulating the biomineralization process. Robinson *et al*. found that spherical mineral particles with a diameter of 50 nm organize into chains during the formation of enamel^17^. It was hypothesized that these nanoparticles are composed of amorphous calcium phosphate (ACP) and proteins associated with biomineralization^18^,^19^. The subsequent proteolytic cleavage of the proteins by proteinases triggers the fusion of the ACP nanoparticles and mineral phase transformation into HAP^20^. In particular, of these proteins, amelogenin is best documented for its roles in the regulation of the biomineralization and structural organization of enamel. Specifically, it is hypothesized that the charged C telopeptides could form an equatorial band that shows a high density of hydrophilic and charged characteristics inside the double-barrel structured oligomers of recombinant full-length murine amelogenin rM179^21^. Thus, the interior of the oligomers can stabilize ACP nanoparticles via electrostatic interactions; these transiently stable mineral nanoparticles can then assemble into linear chains, arrange in close proximity and subsequently fuse and transform into needle-shaped HAP crystals that organize as parallel arrays. This mineralization pattern *in vitro* is similar to that of the development of HAP crystals in enamel found by Robinson *et al*.^22^. The mineralization process mediated by the stable amorphous mineral phases is known as the non-classical crystallization pathway^23^,^24^. These findings provide unique understandings of the regulation of biomineralization by organic macromolecules and are an inspiration for the remineralization of demineralized enamel based on bottom-up strategies.

Based on the understandings of the biomineralization of dental hard tissues, finding analogues of related proteins such as amelogenin that are capable of stabilizing and guiding ACP to assemble in an orderly manner is an important strategy for the development of novel biomimetic-remineralizing agents. Recently, PASP (polyaspartic acid) and PAA (polyacrylate acid) have been used to stabilize ACP nanoparticles in solution due to their rich abundance of carboxyl groups for chelating calcium ions^25^,^26^,^27^,^28^. We also found that carboxymethyl chitosan (CMC), a derivative of chitosan that is rich in carboxyl groups, can facilitate the stabilization of ACP nanoparticles as nanocomplexes of CMC/ACP in solution because of its chelating capacity. However, due to the lack of an affinity for the enamel surface and self-assembly ability, these ACP nanoparticles cannot efficiently adsorb on the surface of enamel and transform into the oriented and ordered HAP crystals to remineralize enamel. More recently, it has been demonstrated that carboxyl-terminated poly(amido amine) (PAMAM-COOH) can promote the HAP crystallization process and thus has potential for the remineralization of dental hard tissues^29^. Furthermore, alendronate (ALN) is conjugated on PAMAM-COOH, causing increased binding strength between PAMAM-COOH and the enamel surface due to its specific adsorption on HA (the primary component of tooth enamel)^30^. Thus, ALN could enhance the binding strength between remineralizing agents and the surface of dental hard tissues. Moreover, it has been reported that both 10 mM glycine and 1.25 μM amelogenin could guide nanospheres with HAP cores and ACP shells (HAP@ACP) that are prepared by partially removing stabilizing agents for ACP into oriented and ordered arrays, finally resulting in bundles of enamel-like HAP crystals by fusion of these nanospheres^31^. According to the results of molecular dynamic simulations, HAP (001) faces tend to be unstable in Gly solutions, and thus, the ordered assembly of HAP crystals along the c-axis is favorable for minimizing the total interfacial energy^32^. The ACP, linking the HAP nanoparticles, started a phase transformation process. During the phase transformation, the “hard-soft-hard” (HAP-ACP-HAP) connections disappeared and the “hard-hard” (HAPtransferred HAP-HAP) attachment began to dominate in the linear assembly with the guiding effect of glycine^31^. It is believed that amino acids or proteins could interact with the HAP over the thin ACP layer^33^,^34^,^35^, which explains why Gly can guide HAP@ACP core-shell nanoparticles into oriented and ordered arrays.

Accordingly, in this study, ALN is conjugated on CMC to enhance its binding ability to enamel. HAP@ACP core-shell nanoparticles are prepared through the degradation of CMC using hypochlorite. Gly is used to guide the nanoparticles into oriented and ordered arrays. Therefore, the hypothesis of this study is that HAP@ACP core-shell nanoparticles could be mediated into oriented and ordered arrays and then absorb to the damaged enamel surface, finally forming well-ordered rod-like apatite crystals to achieve oriented and ordered biomimetic remineralization of dental enamel.

## Results

### NMR of CMC and CMC-ALN

**Figure 1:**
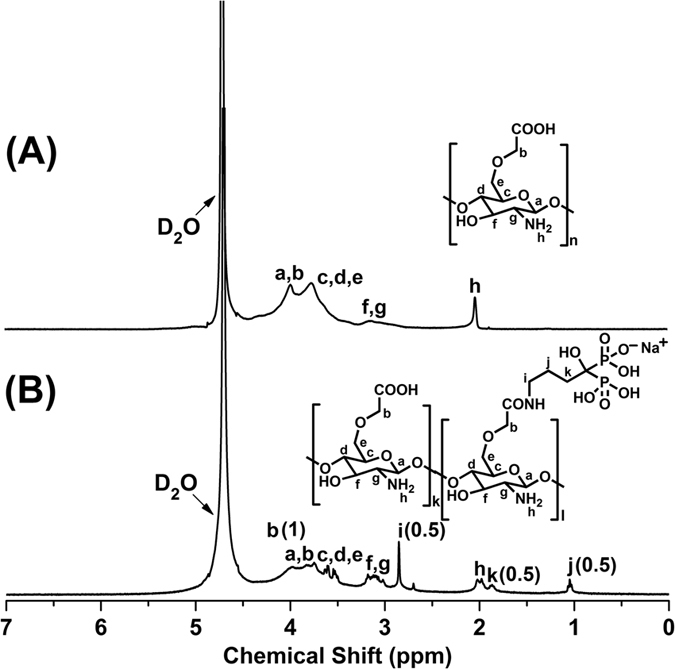
NMR results showing the basic difference between CMC (**a**) and CMC-ALN (**b**): The appearance of new peaks i and j and the change in peaks j, h and k are attributed to the incorporation with ALN.

**Figure 2:**
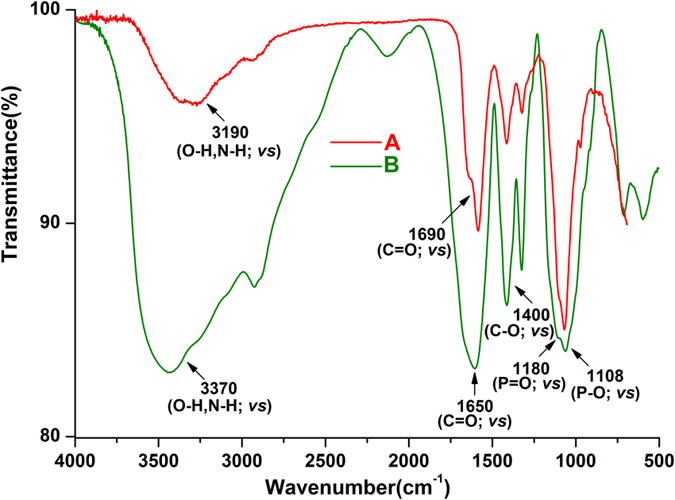
FTIR spectra of (**a**) CMC and (**b**) CMC-ALN; the stretching vibration band changed due to ALN.

The chemical compositions of carboxymethyl chitosan(CMC) and its alendronate(ALN) derivative were determined by 1 H NMR (Fig. 1) and FTIR (Fig. 2) spectroscopic analyses. From the 1H NMR spectra presented in Fig. 1, the most obvious differences are the appearance of the new peaks i and j at 2.8 and 1.1 ppm corresponding to the two methylenes adjacent and interphase to the amino group of the substrate ALN, respectively. Furthermore, peak j splits into a quintet. More importantly, the peaks h and k at approximately 2.0 ppm became more resolved, which is largely attributed to the incorporation of methylene by ALN. All the phenomenon mentioned above indicate the successful linkage of CMC with the amino group of ALN. By comparing the integration areas (in brackets) of i, j and k with b, we can conclude that the graft rate of ALN is 50%.

### FTIR of CMC and CMC-ALN

The successful synthesis of the ALN derivative of CMC can be further verified from the FTIR spectra (Fig. 2). The broad stretching vibration band and strong bending vibration bands of O-H at 3190 cm^−1^ and at 1400 and 960 cm^−1^, respectively, in Fig. 2(a) demonstrate the presence of carbonyl in CMC. Furthermore, the stretching vibration band at approximately 3370 cm^−1^ in Fig. 2(b) became broader and stronger compared to the corresponding section in Fig. 2(a), and it moved to the high-wavenumber area to some extent. This result is largely due to the presence of amino N-H and the incorporated hydroxyl groups from ALN, and it also confirmed that the carboxymethyl groups were connected to the primary hydroxyl rather than the amino groups of the chitosan substrate. The downshift of the C = O stretching vibration band from 1690 cm^−1^ to 1650 cm^−1^, as well as the presence of stretching vibration bands at approximately 1180 and 1108 corresponding to P = O and P-O, indicate the successful linkage of amide.

**Figure 3:**
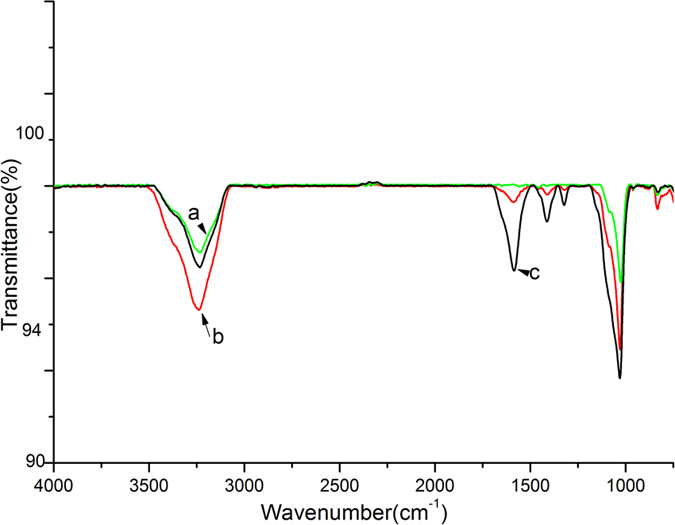
ATR-IR spectra of enamel samples: (**a**) before coating with CMC (pristine enamel surface) showed as green line; (**b**) after coating with CMC followed by washing showed as red line; (**c**) after coating with CMC-ALN followed by washing showed as black line.

Figure 3 presents the ATR-IR spectra of enamel samples before and after coating with CMC and CMC-ALN. In these spectra, the corresponding peak of enamel (1040 cm^−1^ for PO4^3−^) can be clearly observed. Then, after coating with CMC or CMC-ALN, the characteristic peaks of CMC (3500–3050 cm^−1^ for the amide vibration and 1650 cm^−1^ for the amide carbonyl) derived from the amide bonds in its structure appeared in both samples. However, after rinsing with PBS, the characteristic peaks of CMC-coated samples exhibited significant decreases, indicating that the CMC materials on the surface of tooth enamel were easily washed off. In contrast, the CMC ALN-coated sample still presented obvious characteristic peaks.

### TEM

**Figure 4:**
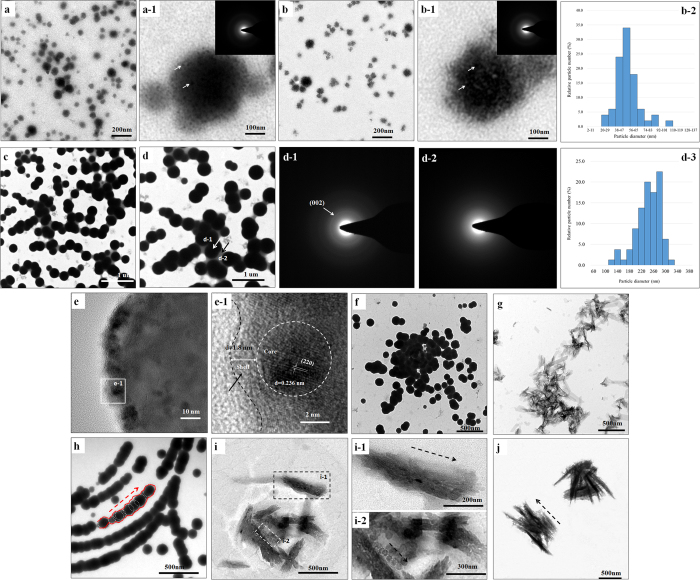
(**a**,**b**) The amorphous nanoparticles of CMC/ACP and CMC-ALN/ACP; (**a-1**) and (**b-1**) the magnified images of representative amorphous nanoparticles (nanocomplexes) in (**a**,**b**) with SAED pattern, respectively. the white arrows indicating smaller particles in the nanocomplexes; (**c**,**d**) the nanoparticles of CMC-ALN/ACP treated with NaClO; (**d-1**) the SAED pattern of area indicated with a white arrow in (**d**); (**d-2**) the SAED pattern of area indicated with a black arrow in (**d**); (**b-2**) and (**d-3**) the frequency distribution bar charts of particles in (**b**,**c**); (**e**) magnification of a representative HAP@ACP nanoparticle; (**f**) disorderly aggregation of HAP@ACP nanoparticles in the absence of Gly; (**g**) formation of mineral crystals with random orientation from (**f**) after 48 h; (**h**) oriented and ordered assembly of HAP@ACP nanoparticles in the presence of Gly; (**i**) the transient state of transformation from HAP@ACP nanoparticles to mineral crystals between (**h**,**j**) after 24 h; (**i-1**) the magnified image of area indicated with a rectangle of black dash line in (**i**), c-axis of crystals indicated by a dash line arrow; (**i-2**) the magnified image of area indicated with a rectangle of white dash line in (**i**), showing the linearly arranged HAP@ACP nanoparticles co-existing with crystals in transient state; (**j**) formation of oriented and ordered mineral crystal bundles from (**h**) after 48 h.

Nanoparticles of CMC/ACP (Fig. 4(a,a-1)) and CMC-ALN/ACP (Fig. 4(b,b-1)) with diameters of less than 200 nm were observed in the TEM images, showing a relatively rough morphology. The obvious dot or ring pattern characteristic of a crystalline structure was not observed in the SAED images (Fig. 4(a-1), which indicates that the main composition of these nanoparticles was an amorphous phase. Figure 4(a-1 show a typical aggregate composed of many smaller nanoparticles; these aggregates could be defined as nanocomplexes.

Following NaClO treatment, the size of the nanoparticles increased and their shape become smooth compared with those of CMC/ACP and CMC-ALN/ACP nanoparticles (Fig. 4(c,d)). The diameters of nanoparticles in Fig. 4b,b have been measured and analyzed. The results have been shown in the frequency distribution bar charts (Fig. 4(b-2), respectively. After the treatment of NaClO, the particle size increased obviously. In Fig. 4(d), the SAED image (Fig. 4(d-1)) corresponding to the core part of the nanoparticles shows an evident ring pattern characteristic, whereas Fig. 4(d-2) corresponding to the part between or around the nanoparticles does not exhibit an obvious structural characteristic, which indicates that after the NaClO treatment, the nanoparticles consist of a crystallized core of HAP and an amorphous layer (i.e., HAP@ACP core-shell nanoparticles). In addition, these nanoparticles were linked by ACP. Moreover, individual units were observed in the HAP@ACP core-shell nanoparticles using HRTEM (Fig. 4(e)). In a representative individual unit, the crystallized HAP cores with a lattice structure and the amorphous shell with a thickness of (1.8 nm could be clearly observed (Fig. 4(e-1)).

With the addition of Gly, the HAP@ACP nanoparticles arranged linearly and tended to fuse together (Fig. 4(h)), whereas without Gly, the HAP@ACP nanoparticles aggregated randomly (Fig. 4(f)). Accordingly, after 48 h, the ordered HAP@ACP nanoparticles transformed into mineral crystal bundles of enamel-like apatite assembling along their c axes (Fig. 4(j)); in contrast, the disordered ones changed into disarranged crystals after 48 h ((Fig. 4(g)). Figure 4(i) and shows the transient state of transformation mediated by Gly after 24 h, in which the linearly arranged HAP@ACP nanoparticles (indicated by a rectangle in Fig. 4(i-2)) co-existed with the mineral crystal bundles (Fig. 4(i-1)).

### SEM

**Figure 5:**
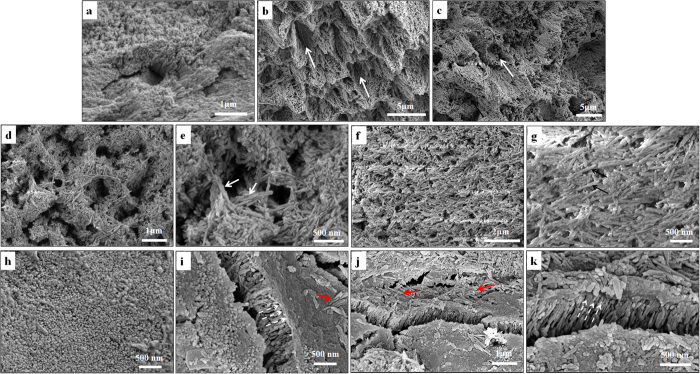
SEM results showing the surface morphology of normal enamel in (**a**). The surface morphologies of demineralized enamel (**b,c**); remineralized enamel with HAP@ACP nanoparticles from CMC/ACP nanocomplexes (**d,e**); remineralized enamel with HAP@ACP nanoparticles from CMC-ALN/ACP nanocomplexes (**f,g**); remineralized enamel with combination of HAP@ACP nanoparticles from CMC-ALN/ACP nanocomplexes and Gly (**h**,**i**,**j**,**k**), showing layers of oriented and ordered mineral crystals perpendicular to the enamel surface indicated with arrows of white dash line, meanwhile, on these layers scattered mineral crystals parallel on the enamel surface indicated with arrows of red dash line.

The surface morphology of the demineralized enamel samples before and after remineralization is shown in Fig. 5. The natural enamel surface was shown in Fig. 5(a). The demineralized enamel surface exhibited a lost enamel prism core but retained periphery (Fig. 5(b)). Ordered or disordered arrangement of the rod-like residual HAP crystallites can be observed in the lesion on the surface of the enamel (Fig. 5(b)). With the remineralizing solution itself (Group A), no evident new crystal formed on the surface of the demineralized enamel (Fig. 5(c)). The HAP@ACP nanoparticles from the CMC/ACP nanocomplexes did not yet lead to abundant newly formed crystals as indicated by white arrows (Fig. 5(d,e)). In contrast, HAP@ACP nanoparticles from CMC-ALN/ACP nanocomplexes caused a layer of newly formed needle-like crystals on the surface of demineralized enamel (Fig. 5(f,g)). These crystals organized randomly and were parallel to the surface, covering the rough and uneven morphology of the sample (Fig. 5(f,g)). In addition, the size of these newly formed crystals was higher than that of residual crystals from the demineralized enamel (Fig. 5(b)) or crystals from normal enamel (Fig. 5(a)). Notably, the combination of the HAP@ACP nanoparticles from CMC-ALN/ACP nanocomplexes and Gly resulted in a layer of an oriented and ordered arrangement of newly formed needle-like crystals (Fig. 5(h,i,j,k)). The c-axis of these crystals was perpendicular to the surface of the enamel, which is similar to the orientation of natural enamel prism.

### Nanoindentation

**Figure 6:**
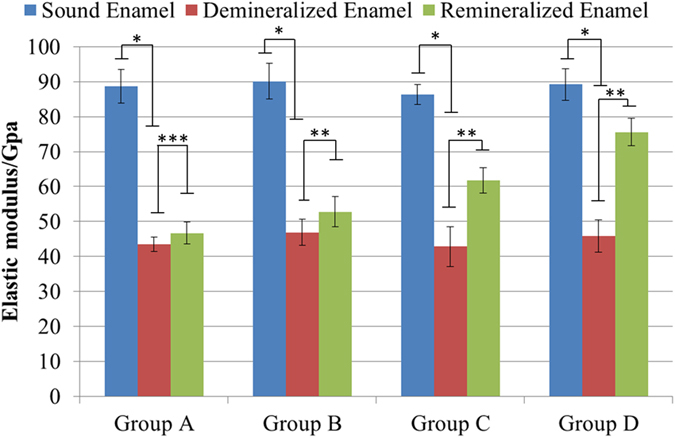
Results of elastic modulus investigation in different remineralization groups.

**Figure 7:**
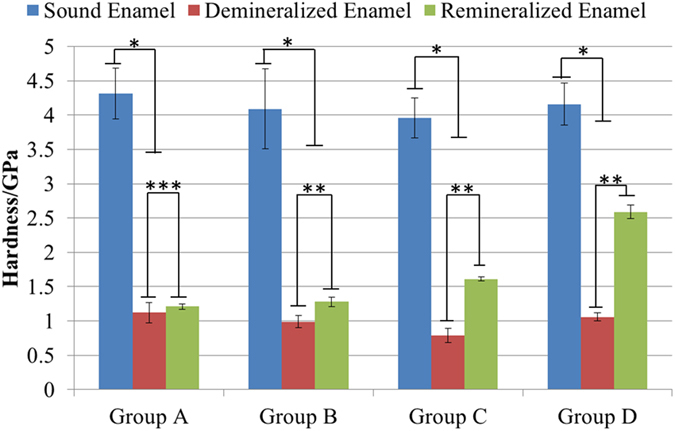
Results of hardness investigation in different remineralization groups.

As shown in Figs 6 and 7, the mechanical properties (i.e., elastic modulus and hardness) of the samples in all groups drastically decreased following the demineralization treatment (P < 0.001). Afterward, with the traditional remineralization treatment, the mechanical properties of the samples in Group A did not significantly recover (P > 0.05) (Figs 6 and 7). In contrast, the different biomimetic remineralization treatments significantly improved the mechanical properties of the samples in Groups B, C and D in various degrees (P < 0.05) (Figs 6 and 7). The recoveries of the elastic modulus and hardness of the samples are shown in Table 1. The HAP@ACP nanoparticles from CMC-ALN/ACP combined with Gly most clearly improved the mechanical properties of the samples.

## Discussion

The essential structure of mature tooth enamel consists of a dense array of hydroxyapatite nanorods with their crystalline c-axes parallel to the nanorods. This oriented and ordered mineralization pattern is highly related to the mechanical properties of enamel, including its hardness and elasticity. Therefore, the *in situ* regeneration of enamel-like structure and components under physiological conditions is significant for the remineralization of demineralized hard tissue of enamel to restore its mechanical properties and physiological function.

**Figure 8:**
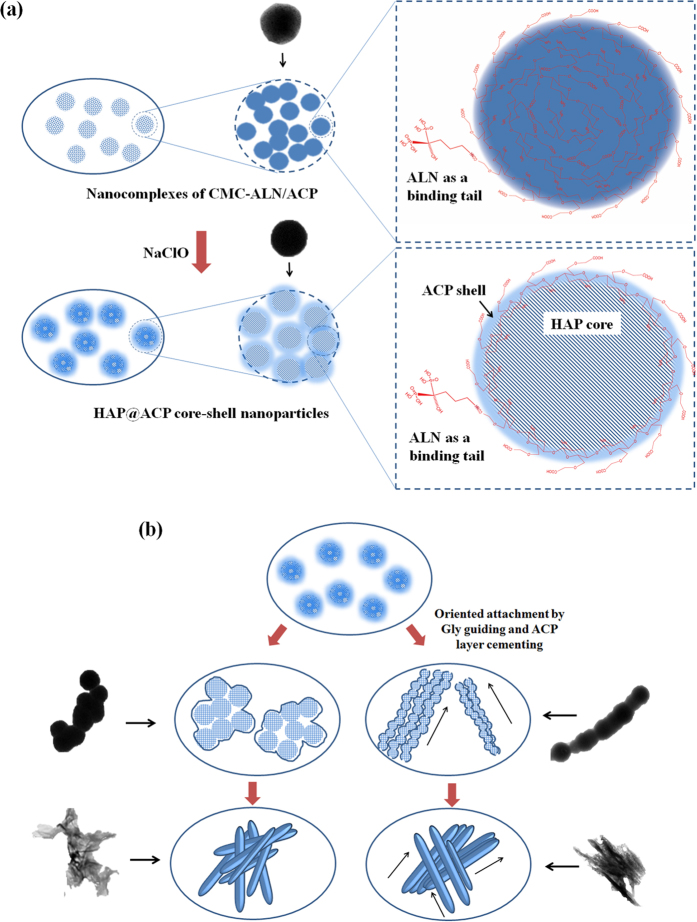
A schematic model of the formation of HAP@ACP nanoparticles from CMC-ALN/ACP nanocomplexes: (**a**) the degradation of CMC-ALN matrix by NaClO causing the formation of HAP@ACP nanoparticles, which are composed of smaller nanoparticles. The ACP layer still containing the CMC-ALN matrix; (**b**) the introduction of Gly leading to the linear arrangement of HAP@ACP nanoparticles and subsequent formation of oriented mineral crystal bundles.

It has been suggested that in the early secretory enamel, the formation of elongated ribbon-like crystals is mediated via the fusion of spherical particles containing ACP and protein^36^,^37^,^38^,^39^. In this study, CMC was used to replace the protein to stabilize ACP by forming nanocomplexes of CMC/ACP (Figs 4(a,b) and 8(a)). In aqueous media, ACP will readily transform into stable crystalline phases because of the growth of a microcrystalline phase. In addition, the affinity of ACP to the enamel surface is extremely low^37^. In this study, nanocomplexes of CMC/ACP also exhibited low affinity to the enamel surface, as indicated by the FTIR results (Fig. 3); thus, these nanocomplexes readily release from the enamel surface, which results in less mineral crystal on the enamel surface.

To enhance the affinity of CMC/ACP nanocomplexes to the enamel surface, ALN was conjugated to CMC in this study due to its strong adsorption to HAP crystals. It was demonstrated that the interaction between ALN and the HAP surface occurs through ligand exchange (i.e., two surface phosphate groups of HAP are replaced by the two phosphate groups of the ALN molecule)^40^. Due to the lack of an affinity for the enamel surface and self-assembly ability, these ACP nanoparticles cannot efficiently adsorb on the surface of enamel and transform into the oriented and ordered HAP crystals to remineralize enamel. While alendronate (ALN) could be conjugated on PAMAM-COOH, causing increased binding strength between PAMAM-COOH and the enamel surface due to its specific adsorption on HA^30^. Thus, ALN could enhance the binding strength between remineralizing agents and the surface of dental hard tissues.

Therefore, ALN has been conjugated to some biological/synthetic polymers as drug carriers to endow them with the special HAP binding ability to develop bone- or tooth-targeted drug delivery systems^41^,^42^,^43^,^44^,^45^,^46^. In this study, ALN was conjugated to CMC to form CMC-ALN; the introduction of ALN did not affect the stabilizing ability of CMC. The morphology of the nanoparticles in nanocomplexes of CMC-ALN/ACP is similar to that of CMC/ACP. In particular, compared with CMC/ACP, more CMC-ALN/ACP specifically adsorbed on the enamel surface, causing significant surface remineralization of demineralized enamel (Fig. 5).

It has been reported that developing enamel presents collinear nanospherical features, as characterized by atomic force microscopy (AFM)^17^,^47^. It has been suggested that these spherical features are crystal precursors that contain calcium and phosphate ions that are associated with perhaps amelogenin, enamelin, and ameloblastin in some manner^17^. In other words, these collinear spheres form a mineral matrix complex as a stabilized amorphous phase as postulated by Posner *et al*.^48^, which even resembles a polymer-induced liquid precursor phase in a way described by Gower *et al*.^49^. In this study, the nanocomplexes of CMC/ACP and CMC-ALN/ACP can mimic amelogenin to stabilize ACP.

It is natural for researchers to question how the stabilized complexes of protein matrix and ACP could transform into a mineral crystal phase. It has been suggested that the transformation is mediated by the rather elaborate protein matrix processing during early enamel development^50^. The initial degradative processing of protein within the complexes of protein matrix and ACP could reduce the binding sites of mineral ion or water content, thereby resulting mineral nuclei^17^. Next, with the further degradation of the proteins, the nuclei would fuse, forming the collinear globular crystal structures^17^. Finally, the protein matrix is completely degraded and replaced by fluid during the maturation stage, and these crystals grow in thickness.

In this study, to mimic the process of proteolytic cleavage of protein matrix, NaClO was used to degrade the CMC or CMC-ALN matrix. Proteins are fragmented in low concentrations of sodium hypochlorite, which could be attributed to the chlorination of amide nitrogen followed by protein oxidation to an imine with further hydrolysis, thus denaturing proteins^51^. CMC, a derivative of chitosan, can also be degraded by NaClO based on its strong oxidizing ability that can attack β-(1,4) glucosidic bonds between molecules to depolymerize CMC with high molecular weight^52^.

With degradation of the CMC or CMC-ALN matrix, the core-shell structure of HAP@ACP nanoparticles could form. In this study, a low concentration of NaClO resulted in partial degradation of the organic matrix. As shown in Fig. 4, the internal structure of the nanoparticles transformed from a comparatively loose (amorphous) to a closely packed (crystalline) structure, with an increase in radius. Without chelating agents, the calcium and phosphate ions in the core of nanoparticles will reorganize, facilitating the internal structure of amorphous particles into ordered crystalline states, thereby acting as a nucleus for the growth of crystalline HAP^52^. In contrast, the organic matrix remained around the core of nanoparticles to stabilize ACP, forming the ACP shell surrounding the HAP core (Fig. 4(e,e-1)). This process is based on non-classical crystallization theory (pathway), in which inorganic nanocrystals coated/stabilized with organic molecules can form larger mesocrystals as shown in Fig. 4(h,i) via self-assembly and crystallographic alignment^53^. The mesocrystals (Fig. 4(h,i,i-1) work as intermediates for the formation of single macroscopic crystals. In this study, without NaClO treatment, the nanocomplexes of CMC/ACP or CMC-ALN/ACP can remain stable at least for one week. Therefore, the utilization of NaClO can improve the speed of the transformation from ACP into HAP, thereby achieving a relatively rapid rate of remineralization, which is significant for clinical applications.

**Figure 9:**
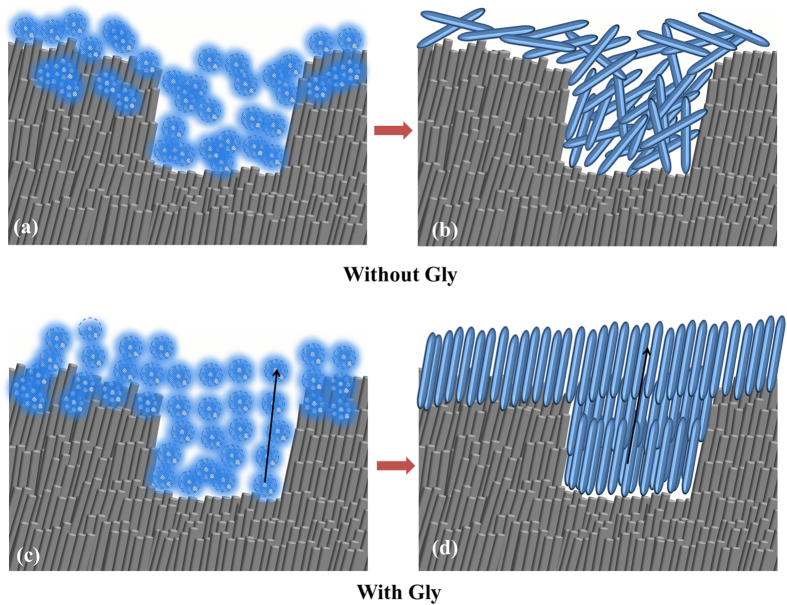
The mechanism diagram illustrating the oriented and ordered remineralization of demineralized enamel. (**a**,**b**) In the absence of Gly, the HAP@ACP nanoparticles only result in the formation of HAP crystals on the sample surface with random orientations; (**c**,**d**) in the presence of Gly, HAP@ACP nanoparticles transform into well-ordered rod-like HAP crystals being perpendicular to the sample surface, recovering the mechanical properties of demineralized enamel.

It has been established that Amel-ACP nanoscale building blocks are spontaneously formed by synergistic interactions between flexible Amel protein molecules and Ca-P prenucleation clusters, and these spherical nanoparticles evolve by orientated aggregation to form nanochains. This process is based on the non-classical mechanism of crystal growth, oriented attachment (OA), which is conducted by the attachment events between specific crystal faces that are lattice-matched, either with true crystallographic alignment or across a twin boundary or stacking fault^54^. Structured macromolecules such as amelogenin can promote the OA process^55^. However, CMC or CMC-ALN itself cannot guide ACP nanoparticles into an oriented and ordered array. HAP@ACP nanoparticles from nanocomplexes of CMC-ALN/ACP cannot spontaneously connect and fuse orderly, only showing random aggregates (Fig. 8(b)). In contrast, the introduction of Gly caused the linear arrangement of HAP@ACP nanoparticles (Fig. 8(b)). The oriented attachment of HAP@ACP nanoparticles promoted by Gly could be attributed to the anisotropic change in interfacial energies (γ) of HAP solution caused by Gly at the different crystallographic directions, as demonstrated by computer simulations.52 In the absence of Gly, the γ of (001) face is lower than that of the (100)/(010) face, whereas the introduction of Gly reverses this relationship by greatly increasing the γ of the (001) face and by slightly reducing that of the (100)/(010) face^31^,^32^. Because it is difficult to form such an interface between the solid and aqueous phases, the (001) faces of HAP became unstable in the presence of Gly, resulting in the ordered assembly of HAP crystals along the c-axis, which is preferred for minimizing the total interfacial energy of the assembly in the solutions^31^,^32^. Notably, it was demonstrated that the interaction between amino acids and HAP crystal faces could be achieved over a thin layer with a distance of 1.0 nm^56^ through computer simulation. It was found that a continuous layer of amorphous CaCO_3_ (ACC) covers the aragonite CaCO_3_ platelets in the nacre of Haliotis laevigata^33^. This 3- to 5-nm ACC carbonate layer does not affect the charge interaction of the highly polar aragonite face^57^. In the core/shell structure of HAP@ACP nanoparticles, the thickness of the ACP shell was at the same scale (Fig. 4(e-1)), and the crystal faces of HAP core were also highly polarized. Thus, it was reasonable that Gly interacted with the HAP over the thin ACP layer. Recently, the classical nucleation theory has been used to explain the remineralizing mechanism of ACP derivatives such as CPP-ACP^58^ and Pchi-ACP^2^. That is, they work as a source of calcium and phosphate ions to remineralize enamel lesions via ion-by-ion addition to the nucleation sites or pre-existing seed crystallites^32^,^59^,^60^. In contrast to these crystallization pathways of monomer-by-monomer addition, crystallization by addition and oriented attachment of particles, ranging from multi-ion complexes to fully formed nanocrystals, is now well recognized^55^. In this study, the TEM results (Fig. 4) support the mineralization pathway based on the conception of crystallization by particle attachment (CPA)^55^. The thin ACP layer cementing nanoparticles and Gly guiding nanoparticles contribute to the oriented attachment of HAP@ACP nanoparticles. Based on this theory, ordered arrays of HAP@ACP nanoparticles as mesocrystals are able to finally transform into oriented and ordered arrays, but disordered ones could not. The remineralization process observed with HAP@ACP nanoparticles from CMC-ALN/ACP is schematically summarized in Fig. 9. This process is similar to that of the biomineralization of human enamel, in which the co-assembly of amelogenin and ACP clusters initially occurs followed by transformation into the crystalline phase with degradation of amelogenin caused by metalloendoprotease^20^,^61^,^62^. Only the combination of the HAP@ACP nanoparticles from CMC-ALN/ACP and Gly can lead to the formation of ordered HAP crystals being perpendicular to the surface of demineralized enamel, which is consistent with the orientation of natural HAP crystals of enamel (Fig. 9(c,d)). Without Gly, only disordered HAP crystals being parallel to the surface of the samples formed (Fig. 9(a,b)). Clearly, the recovery rates of the mechanical properties of demineralized enamel are highly related to the orientation of newly formed crystals, which will be further investigated in the future. Accordingly, the assembled structure and pattern of newly formed HAP crystals resulting from the combination of the HAP@ACP nanoparticles from CMC-ALN/ACP and Gly are similar to the natural one, that is to say, HAP@ACP core-shell nanoparticles could be mediated into oriented and ordered arrays and then absorb to the damaged enamel surface and finally formed well-ordered rod-like apatite crystals to achieve oriented and ordered biomimetic remineralization of dental enamel. The oriented and ordered remineralization can significantly recover the mechanical properties of demineralized enamel.

Figure 5(h,i) showing relatively clearly oriented and ordered mineral crystals perpendicular to the enamel surface. In Fig. 5(i,j), both oriented and ordered mineral crystals perpendicular to the enamel surface and those parallel on the enamels surface indicated by red arrows were found. Although ideal oriented and ordered mineralization pattern was not accomplished, it is should be noted that the mineral crystals parallel on the enamel surface only formed on the layer of oriented and ordered mineral crystals, which did not affect the mechanical behavior of the oriented and ordered mineralization pattern (Figs 6 and 7). The formation of these mineral crystals parallel on enamel surface could be attributed to the secondary crystallization. The methodology of biomimetic mineralization to avoid the formation of mineral crystals parallel on enamel surface will be further investigated in future.

Since it is difficult to mimic the natural white spot caries showing the irregular shape of the destruction *in vitro*, acid-etched model was made in this study to obtain enamel samples with relatively regular and controllable morphology. In addition, according to the clinical treatment to enamel caries, the strongest binding effect was created by using 30–40% phosphate acid for at least 30 s before coated with binding agent, which will made the most exposure of the enamel rods and wipe out the bacteria biofilm^63^. Therefore, for further clinical use of our remineralization product, an acid-etched surface of the early caries is required because: 1. Unify the shape of the different caries and expose more enamel; 2. Wipe out biofilm and clean the operation surface; 3. the sound enamel surface is beneficial to the connection between alendronate and hydroxyapatite, facilitating the special adsorption of CMC-ALN onto the enamel surface.

## Methods

The institutional review board of the Tianjin Medical University approved the all the procedure and methods in the experiments conducted in this study (TMUh-MEC2012019). All the methods performed in this study in accordance with the relevant guidelines and regulations of Tianjin Medical University.

### Synthesis of CMC-ALN

One-hundred milligrams of CMC (95%, Qingdao Hong Hai Biological Technology Co., LTD, Qingdao, China) was dissolved in 20 ml of 2-(N-morpholino) ethanesulfonic acid (MES: 99%, TCI Chemical Industry Co., LTD, Tokyo, Japan) water buffer in a 50 ml beaker. Then, 20 mg of 1-ethyl-3-(3-dimethylaminopropyl) carbodiimide (EDC: 95%, Sigma-Aldrich Inc., St. Louis, MO, USA) was added to the CMC solution under stirring (300 rpm) for 30 min at 25 °C; 11 mg of N-hydroxysuccinimide (NHS: 97%, Sigma-Aldrich Inc., St. Louis, MO, USA) was gradually added and reacted for 30 min. Next, 60 mg of ALN was added to the reaction solution under stirring (300 rpm) for 12 h. Finally, the solution was dialyzed against water (molecular weight cut-off of 3500) for 48 h at room temperature, and the solution was lyophilized to obtain the CMC-ALN powder. The yield of CMC-ALN was 50%.

### Preparation of nanocomplexes of CMC-ALN/ACP and HAP@ACP core-shell nanoparticles

First, 6.96 mg of potassium hydrogen phosphate (K_2_HPO_4_: Sangon, Biotech, Shanghai China) and 11.76 mg of calcium chloride dehydrate(CaCl_2_ 2H_2_O: Sangon, Biotech, Shanghai China) were added to duplicate 10 ml CMC-ALN or CMC solutions (5 mg/ml) under stirring (500 rpm) for 10 min, respectively. Next, the solution containing CaCl_2_ was added dropwise to the solution containing K_2_HPO_4_ under stirring (500 rpm) for 10 min to form nanocomplexes of CMC-ALN/ACP. The final concentrations of calcium and phosphate in the solution were 4 mM and 2 mM, respectively. The solution of nanocomplexes of CMC-ALN/ACP was stored in a refrigerator at 4 °C.

Prior to the remineralization treatment, 1 ml of 5% (w/v) sodium hypochlorite (NaClO) was added dropwise to a 20 ml solution of nanocomplexes of CMC-ALN/ACP or CMC/ACP under stirring (500 rpm) for 10 min to prepare HAP@ACP core-shell nanoparticles via the partial degradation of CMC-ALN or CMC. Then, 22.5 mg of Gly was added dropwise to a 20 ml solution of HAP@ACP core-shell nanoparticles to guide the nanoparticles. After 30 min, the HAP@ACP and HAP@ACP/Gly core-shell nanoparticles were characterized using TEM.

### Preparation of artificial enamel caries

Before we collected the teeth, the written informed consent was shown to the participants and eventually obtained from all of the subjects. Please include this statement in the methods section. With removal of roots and pulps, these teeth were sawn from the buccal and lingual surfaces to obtain enamel slabs. Twenty-four enamel blocks of approximately 4 × 4 mm^2^ (length × width) were prepared and sealed with water-resistant nail varnish, except for a window (3 × 3 mm^2^) for demineralization and remineralization treatments. Prior to the demineralizing treatment, all enamel samples were sonicated for 30 min. Subsequently, samples of demineralized enamel were prepared by coating a gel of 37% phosphoric acid (Gluma(Gel, Heraeus Kulzer) on the surface of the enamel for 60 s. The samples were then washed with sterile DI water for 60 s, sonicated again for 5 min and stored at 4 °C in sterile DI water prior to use.

### Remineralization of demineralized enamel

The traditional remineralization treatment was performed by immersing enamel samples (Group A, n = 6) into remineralizing solution (10 ml/sample). The biomimetic remineralization treatments were performed by coating HAP@ACP core-shell nanoparticle solutions (100 μl), namely, CMC/ACP (Group B, n = 6), CMC-ALN/ACP (Group C, n = 6) and CMC-ALN/ACP + Gly (Group D, n = 6), onto the window area of the samples using disposable micro applicators (TPC, Advanced Technology, USA). Then, the samples were immersed into the remineralizing solution (10 ml/sample) mentioned above to simulate an oral environment favorable for remineralization. During the 7-day remineralization process, the remineralizing solution was changed every day, and the pipetting of HAP@ACP core-shell nanoparticles was performed daily.

### Characterization of CMC-ALN and its binding ability to enamel surface

FTIR spectra of CMC and CMC-ALN powders were recorded in transmission mode using the KBr pellet method. Spectra were collected in the range from 400 to 4000 cm^−1^ at a 16 cm^−1^ resolution with 100 scans using an infrared spectrophotometer (Shimadzu 8400S, Tokyo, Japan). 1H NMR spectra of CMC and CMC-ALN were recorded in D2O using a Bruker AVANCE III 400 MHz NMR spectrometer at 25 °C operating at 400 MHz and 100.6 MHz, respectively. Tetramethylsilane was used as the internal reference.

The window areas of the enamel samples were evenly coated with 100 μl of CMC or CMC-ALN (5 mg/ml) using disposable micro applicators (TPC, Advanced Technology, USA) and dried at room temperature. Each sample was rinsed three times with deionized water and dried again. ATR-FTIR characterization was conducted in reflection mode using an infrared spectrophotometer (Shimadzu 8400 S, Japan), and spectra were collected in the range from 720 to 4000 cm^−1^ at a 16 cm^−1^ resolution with 100 scans.

### TEM characterization of nanocomplexes of CMC-ALN/ACP or CMC/ACP and HAP@ACP core-shell nanoparticles

The sizes and morphologies of the nanocomplexes of CMC-ALN/ACP or CMC/ACP and HAP@ACP core-shell nanoparticles were characterized using transmission electron microscopy (TEM) (JEM-1230, JEOL, Tokyo, Japan) and selected-area electron diffraction (SAED) at 110 kV. Specimens for the TEM investigation were prepared by slowly evaporating a drop of sample solutions at room temperature on a 400 mesh copper grid covered by a carbon support film.

### Nanoindentation test

Before being undergone the SEM and XRD characterization, all samples were tested using a commercial nanoindenter (Agilent Nano Indenter G200; Agilent, USA) equipped with a Berkovich tip. The machine conformed to the ISO14577 standard. The experiments were conducted at a constant load of 100 mN, and the loading times were fixed at 15 seconds. The load process consisted of loading approach to the surface (at 10 nm/s), holding time at peak load (10 s), and an unloading segment (at 10 nm/s). The nanoindenter machine comes with Agilent NanoSuite 6.1 Professional software. The nanohardness and elastic modulus were evaluated from the load versus depth curves.

The degree of elastic modulus recovery was calculated as R_e_ = 100 × (E_r_ − E_d_)/(E_s_ − E_d_) (E_s_, elastic modulus of sound enamel; E_d_, elastic modulus of demineralized enamel; and E_r_, elastic modulus of remineralized enamel). The degree of hardness recovery was calculated as R_h_ = 100 × (H_r_ − H_d_)/(H_s_ − H_d_) (H_s_, hardness of sound enamel; H_d_, hardness of demineralized enamel; and H_r_, hardness of remineralized enamel).

The mean hardness and reduced elastic modulus values derived from the three indentations performed on each sample were calculated for each group of six samples. Because it was found that both the hardness and reduced elastic modulus data were not normally distributed (Kolmogorov–Smirnov test), a Kruskal–Wallis test combined with a box-whisker plot with 95% confidence interval notches was employed in this study to identify groups of significant difference.

### SEM characterization of biomimetic remineralization of demineralized enamel

The changes in the surface morphology of the enamel samples before and after the remineralization treatments were characterized by scanning electron microscopy (JSM-5600 LV, JEOL, Japan) using a beam voltage of 15 kV and field emission gun scanning electron microscope (JSM-6701F, JEOL, Japan). The enamel samples were first dehydrated using ethanol (at gradient concentration of 70%, 80%, 90% and 100% for 20 min) and then immersed in hexamethyldisilazane, which was allowed to evaporate slowly as the final chemical dehydration step. Subsequently, the enamel samples were sputter-coated with gold prior to SEM characterization.

### Statistical analysis

The data of experiment were evaluated using a two-way ANOVA at a 95% confidence level, with the factors being exposure time and solution (remineralizing solution or mineral water). A multiple range test (Fisher’s least significant difference (LSD) procedure) at a 95% confidence level was performed to identify statistically homogeneous groups.

## Conclusions

With the mediation of Gly, HAP@ACP nanoparticles modified with ALN can arrange orderly and further form well-ordered rod-like apatite crystals to achieve the oriented and ordered biomimetic remineralization of enamel. This would be a promising method for the management of early caries in minimal invasive dentistry. This study indicates that finding and developing analogues of natural proteins such as amelogenin involved in the biomineralization of enamel from natural macromolecular polymers and imitating the process of biomineralization would be an effective strategy for enamel remineralization.

## Additional Information

**How to cite this article**: Wang, H. *et al*. Oriented and Ordered Biomimetic Remineralization of the Surface of Demineralized Dental Enamel Using HAP@ACP Nanoparticles Guided by Glycine. *Sci. Rep.* **7**, 40701; doi: 10.1038/srep40701 (2017).

**Publisher's note:** Springer Nature remains neutral with regard to jurisdictional claims in published maps and institutional affiliations.

## Figures and Tables

**Table 1:** Elastic modulus and hardness recovery percentages of different remineralization groups.

	Group B	Group C	Group D
Re	13.7%	43.4%	68.6%
Rh	9.4%	25.9%	49.4%
